# A novel strategy to engineer pre-vascularized 3-dimensional skin substitutes to achieve efficient, functional engraftment

**DOI:** 10.1038/s41598-019-44113-6

**Published:** 2019-05-24

**Authors:** Hiromi Miyazaki, Yasuyuki Tsunoi, Takami Akagi, Shunichi Sato, Mitsuru Akashi, Daizoh Saitoh

**Affiliations:** 1 Division of Traumatology, Research Institute, National Defense Medical College, 3-2 Namiki, Tokorozawa, Saitama 359-8513 Japan; 2 Division of Bioinformation and Therapeutic Systems, Research Institute, National Defense Medical College, 3-2 Namiki, Tokorozawa, Saitama 359-8513 Japan; 3 Graduate School of Frontier Biosciences, Osaka University, 1-3 Yamadaoka, Suita, Osaka 565-0871 Japan

**Keywords:** Implants, Tissue engineering

## Abstract

Autologous split-thickness skin grafts are the preferred treatment for excised burn wounds, but donor sites for autografting are often limited in patients with extensive burns. A number of alternative treatments are already in use to treat large burns and ulcers. Despite intense efforts to develop tissue-engineered skin, delayed or absent vascularization is one of the major reasons for tissue-engineered skin engraftment failure. To overcome these problems, we developed a scaffold-free 3-dimensional (3D) skin substitute containing vascular networks that combine dermal fibroblasts, endothelial cells, and epidermal keratinocytes based on our layer-by-layer cell coating technique. We transplanted the pre-vascularized 3D skin substitutes onto full-thickness skin defects on severe combined immunodeficiency mice to assess their integration with the host tissue and effects on wound healing. We used non-vascularized 3D skin substitutes as a control. Vessels containing red blood cells were evident in the non-vascularized control by day 14. However, blood perfusion of the human-derived vasculature could be detected within 7 days of grafting. Moreover, the pre-vascularized 3D skin substitutes had high graft survival and their epidermal layers were progressively replaced by mouse epidermis. We propose that a novel dermo-epidermal 3D skin substitute containing blood vessels can promote efficient reconstruction of full-thickness skin defects.

## Introduction

Skin defects caused by severe burns, trauma, or non-healing chronic wounds are common and cause clinical problems. Although numerous approaches and skin substitutes have been developed, the current “golden standard,” consisting of split-thickness skin autografts or skin flap transplantation, is still considered the safest, most effective, and most economically viable method to achieve permanent wound coverage and healing^1,2^. However, for skin injuries involving an extended surface area, limited donor sites are available from which to harvest autografts. In addition, split-thickness skin autografts without dermal support are prone to wound contraction and scar formation^3^.

Sufficient oxygen and nutrition supply and wound infection prevention are critical to the healing process. These factors rely on vascularization, which is a key process in skin tissue engineering that determines the biological function of artificial skin grafts. Insufficient vascularization may result in infection, partial necrosis, or even complete loosening of implanted skin substitutes^4,5^. Hence, efficient vascularization is a major prerequisite for the safe application of these grafts in clinical practice. Recently, many attempts have been made to develop an ideal skin substitute that accelerates wound healing via fast ingrowth of new vessels^1^. In addition to modification of the structural and physicochemical properties of dermal scaffolds, current pre-vascularization approaches may include emerging strategies, such as the incorporation of adipose tissue-derived microvascular fragments^6^ or induced pluripotent stem cell (iPSC)-derived endothelial cells^7^ into the dermal component, three-dimensional (3D) bioprinting^8^, and cell sheet engineering^9,10^. However, except for the 3D printing approach, engineering of the blood vasculature within the dermal matrix requires up to 3 weeks. In addition, most of these techniques are not sufficient to induce high engraftment rates. Therefore, a new strategy with better therapeutic effects is still needed.

Our group recently developed a method for the rapid construction of multicellular stratified 3D tissues by cell surface coating with a nanometer-scale extracellular matrix (ECM) film composed of fibronectin and gelatin (FN-G) using layer-by-layer (LbL) assembly^11,12^. This technique enables the creation of 3D constructs without exogenous scaffolds or biomaterials because the ECM film, similar to the natural ECM, promotes cell-to-cell interaction and adhesion. Moreover, we reported the fabrication of pre-vascularized, functional 3D tissue constructs by coculturing FN-G-coated fibroblasts, hepatocytes, or iPSC-derived cardiomyocytes with vessel-forming cells, such as tissue-derived microvascular endothelial cells^13–16^. Recently, we developed a dermo-epidermal skin equivalent *in vitro* with characteristics, such as permeability to chemicals, similar to those of human skin^17,18^. Therefore, our engineering technique, which creates a vascular network within the multicellular stratified tissues, may have many advantages over conventional approaches for the fabrication of functional, transplantable tissues for tissue replacement.

The objectives of the present study were to generate a scaffold-free pre-vascularized dermo-epidermal 3D skin substitute and to evaluate its therapeutic efficacy in the mouse full-thickness excisional wound-splinting model. Here, we developed a novel 3D skin substitute containing blood vessels using a FN-G ECM film coating technique. The grafted substitutes rapidly anastomosed with host vasculature, exhibited long-term survival and promoted skin regeneration *in vivo*. Our study provides that pre-vascularized LbL 3D skin be a promising substitute for the clinical applications in the future.

## Results

### Characteristics of scaffold-free pre-vascularized 3D skin substitutes

**Figure 1:**
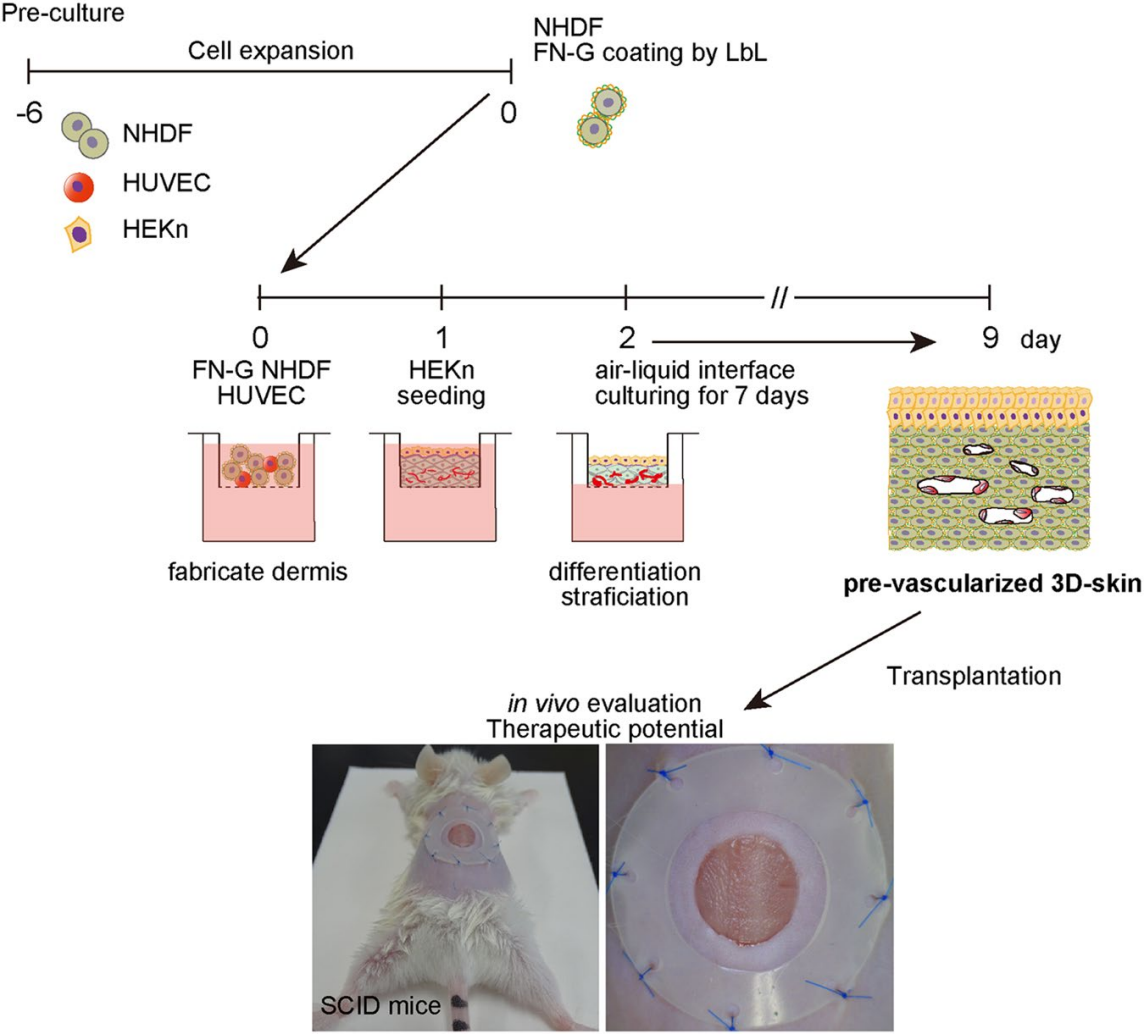
Pre-vascularization of scaffold-free 3D skin. Schematic drawing of the general strategy for creating vascularized dermo-epidermal skin substitutes *in vitro*. NHDF: neonatal normal human dermal fibroblasts; HUVEC: human umbilical vein endothelial cells; HEKn: neonatal human epidermal keratinocytes; FN-G: fibronectin and gelatin; LbL: layer-by-layer.

**Figure 2:**
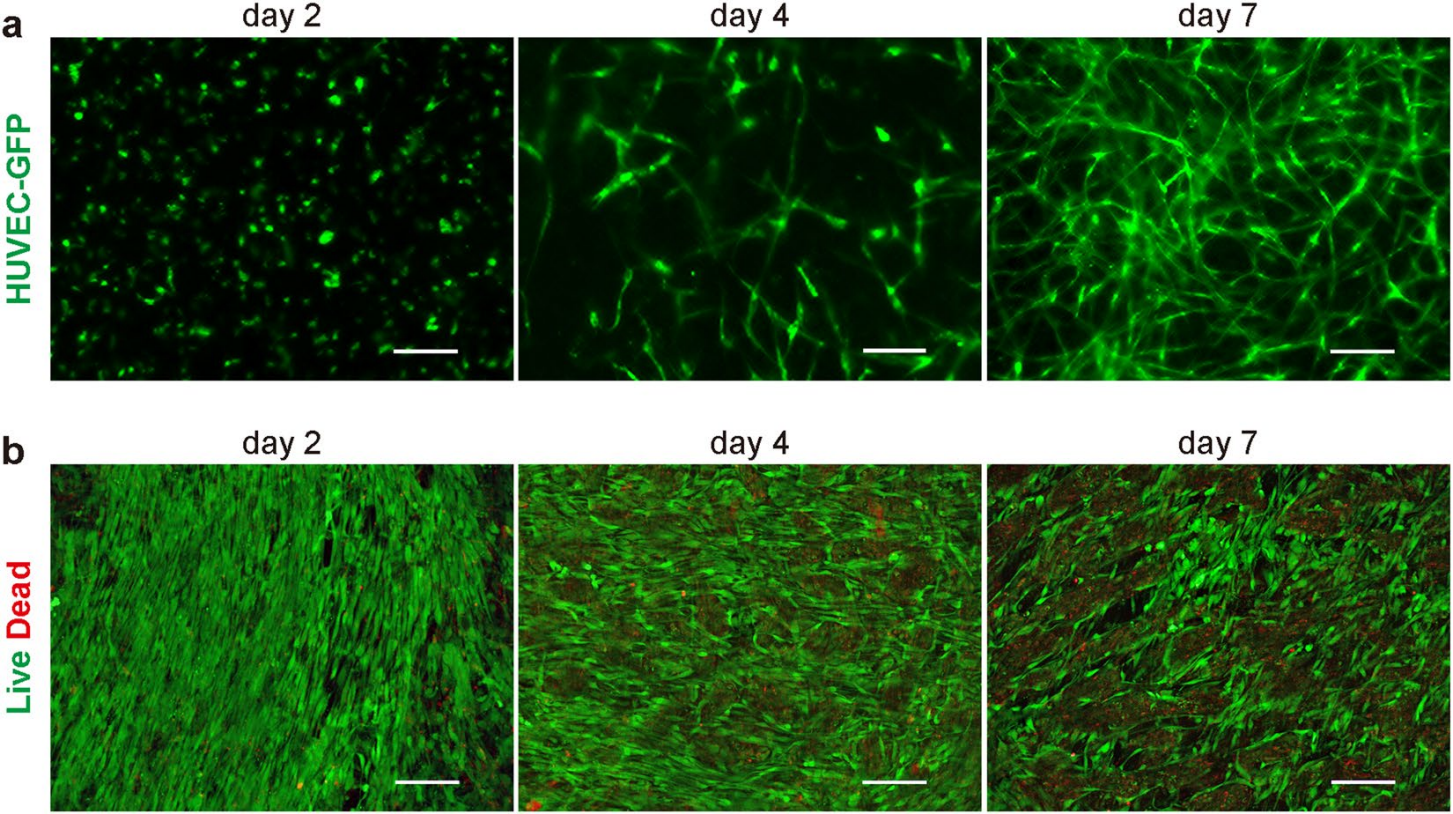
*In vitro* vessel network formation in dermo-epidermal 3D skin substitutes during cultivation. (**a**,**b**) GFP-expressing HUVEC or HUVEC (1 × 10^5^ cells/insert) mixed with FN-G-coated NHDF (1 × 10^7^ cells) were cocultured, subsequently covered with HEKn (1 × 10^6^ cells), then cultured for up to an additional 7 days. (**a**) The dynamics of vessel-like formation in organotypic cocultures. Representative images of vascular network formation within 3D skin after air–liquid interface cultivation. (**b**) The viability of the cocultured cells in the 3D skin as assessed by LIVE/DEAD assay. Representative images (z-stack; 20 μm) of cultured cells (live: green; dead: red) in constructs on days 2, 4, and 7. Scale bars: 100 µm. The data are representative of 3 independent experiments.

Over approximately 15 days of cell culture, including cell expansion periods, we fabricated pre-vascularized dermo-epidermal 3D skin substitutes without using exogenous scaffolds or biomaterials (Fig. 1). In our system, GFP-expressing HUVEC demonstrated elongated morphology and multicellular structures within the dermal layer by day 4 of cultivation. Over the following days, the vessel-like structures increased in number and length (data not shown). By day 7, mature vessels with lumens were visible (Fig. 2a). In our experiments, cells were cocultured at high density (>1 × 10^7^ cells/cm^2^) to construct scaffold-free 3D skin substitutes. We evaluated the constructs in cell viability assays. Compared to the number of green-stained viable cells, there were relatively few red-stained non-viable cells during cultivation. Although a few dead cells appeared after 4 days, the number of live cells remained much higher even after 7 days of cultivation (Fig. 2b).

**Figure 3:**
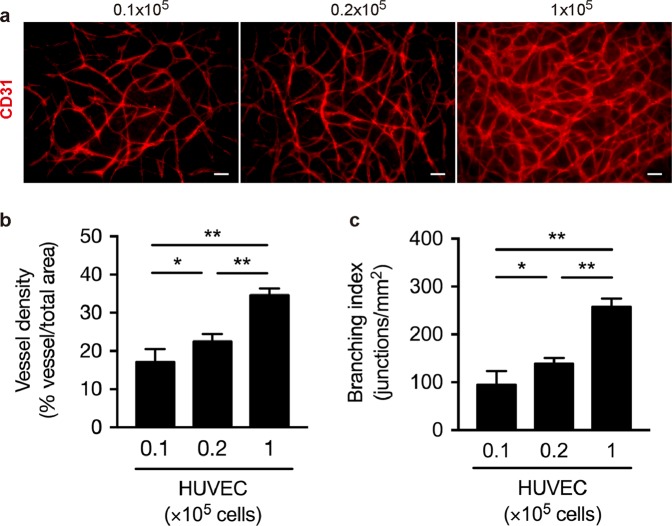
Interconnected vascular network formation in 3D skin substitutes 7 days after epidermal differentiation. (**a**–**c**) HUVEC were seeded at 0.1 to 1 × 10^5^ cells per culture insert; FN-G-coated NHDF content (1 × 10^7^ cells) remained constant. FN-G-coated NHDF and HUVEC were cocultured at 1,000:1, 500:1, or 100:1, and then with HEKn (1 × 10^6^ cells). (**a**) Whole-mount CD31 immunofluorescence analysis revealed that HUVEC developed branching vessels within the dermal layer. The data are representative of 2 independent experiments (n = 6/condition). (**b**,**c**) Quantification of vascular structures to determine the vessel density (**b**) and branching index (branching points/unit area) (**c**). The data are the mean ± SD (n = 6). *p < 0.05 and **p < 0.01 determined by one-way ANOVA with Tukey’s multiple comparison post hoc test.

To evaluate the ratio of HUVEC to FN-G-coated fibroblasts for optimal cell density, we seeded 0.1 to 1 × 10^5^ HUVEC per culture insert while keeping the number of FN-G-coated NHDF constant (1 × 10^7^ cells). Coculture of FN-G-coated NHDF and HUVEC successfully induced the formation of vessel-like networks in the reconstructed dermis consisting NHDF, which varied based on the fibroblast-to-endothelial cell ratio, after 7 days of keratinocyte differentiation (Fig. 3a). The vessel density and branching index (the number of branches per total vessel area) were all maximized by seeding 1 × 10^5^ HUVEC (Fig. 3b,c). On the other hand, vessels did not form in the absence of FN-G-coated NHDF (data not shown).

**Figure 4:**
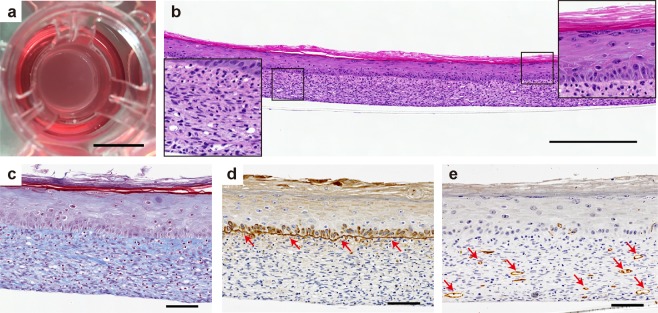
*In vitro* evaluation of the therapeutic potential of pre-vascularized 3D skin substitutes. (**a**–**e**) HUVEC (0.2 × 10^5^ cells/insert) mixed with FN-G-coated NHDF were cocultured, subsequently covered with HEKn, then cultured for up to an additional 7 days. (**a**) Macroscopic view of the construct in the culture insert. Scale bar: 10 mm. (**b**–**e**) Histological and immunohistochemical staining with hematoxylin and eosin (**b**), Masson’s trichrome (**c**), anti-laminin 5 (**d**, arrows = basement membrane), and anti-CD31 (e, arrows = CD31^+^ blood vessel). Scale bars: 500 μm (**b**), 100 μm (**c**–**e**).

To prepare the pre-vascularized 3D skin substitutes for transplantation, HUVEC (0.2 × 10^5^ cells) and FN-G-coated NHDF (1 × 10^7^ cells) were seeded and the surface of the dermal-like layer was inoculated with keratinocytes. Following culture at the air–liquid interface for 7 days, we observed little contraction of the structure of the 3D skin (Fig. 4a). Histological examinations of the pre-vascularized 3D skin substitutes revealed a well-stratified, cornified epidermis and a homogenous dermal component with dense collagen fibers (Fig. 4b,c). Laminin 5, an essential component of the basement membrane structure, was detected in a continuous line at the dermal–epidermal junction after maturation *in vitro* (Fig. 4d). Coculture of HUVEC and FN-G-coated NHDF seeded with keratinocytes for 9 days resulted in the formation of CD31^+^ lumenized vessels within the dermal component (Fig. 4e). The constructs were approximately 320 μm thick with a pre-vascularized dermis of 200 μm.

### Engineered human blood vessels were functional *in vivo*

**Figure 5:**
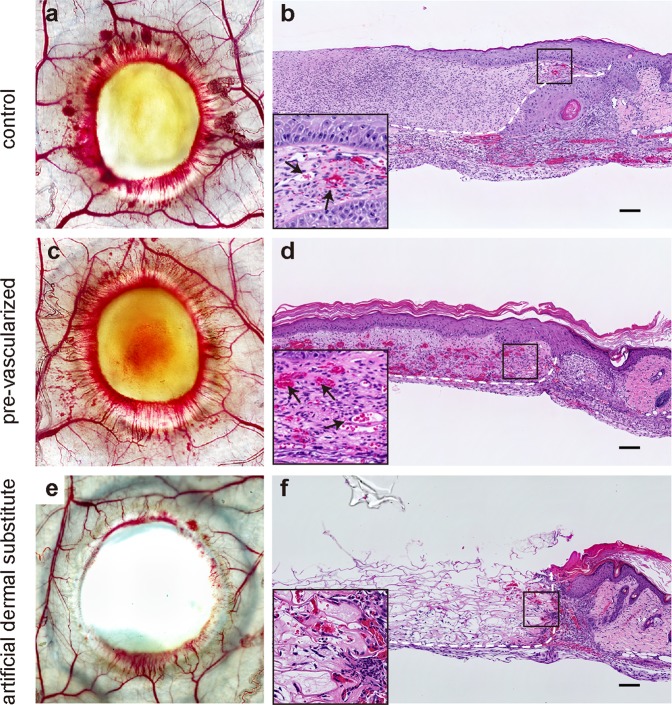
Rapid induction of a functional vasculature in the pre-vascularized 3D skin substitutes *in vivo*. (**a**–**f**) Transillumination microscopy (**a**,**c**,**e**) and hematoxylin and eosin-stained sections (**b**,**d**,**f**) of each substitute 7 days after grafting. Dashed white lines indicate skin substitute–host interface. Black arrows indicate vessels perfused by mouse blood. Scale bars: 100 μm.

**Figure 6:**
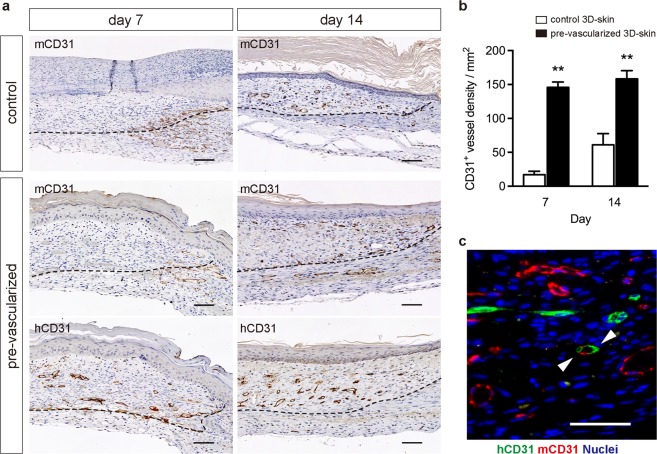
Quantification of wound vasculature *in vivo*. (**a**) Immunohistochemical detection of mouse-specific CD31 (mCD31) and human-specific CD31 (hCD31) at 7 and 14 days after grafting. Dashed lines indicate the skin substitute–host interface. Scale bars: 100 μm. (**b**) Quantification of CD31^+^ blood vessels in the dermal areas of grafts. The data are the mean ± SD (n = 5). **p < 0.01 compared with the non-vascularized control (unpaired Student’s *t*-test). (**c**) Immunofluorescence image stained for mouse-CD31 (red) and human-CD31 (green) of pre-vascularized substitute-treated wounds at day 14 after grafting. Nuclei were stained with DAPI (blue). White arrowheads indicate human–mouse chimeric vessel. Scale bars: 50 μm.

To demonstrate the functionality of the constructed vascular plexuses *in vivo*, we transplanted pre-vascularized 3D skin substitutes onto the excisional full-thickness wounds of immunodeficient mice. On day 7, transillumination analysis revealed that the artificial dermal substitute-treated wounds appeared more transparent than the non-vascularized 3D skin substitutes (control), whereas both control- and pre-vascularized substitute-treated wounds contained blood vessels growing from the surrounding host tissue. In addition, gross hyperemia and more abundant invasion of the vasculature were observed in the wounds treated with pre-vascularized substitutes compared to the wounds treated with the non-vascularized control substitutes (Fig. 5a,c,e). Histological examinations revealed that, as early as 7 days after grafting, the wounds treated with the pre-vascularized substitutes contained blood-perfused vessels (Fig. 5d), whereas the wounds treated with non-vascularized control and artificial dermal substitutes did not contain perfused vessels. In addition, some ingrown blood vessels of host origin were observed in the wound borders at day 7 (Fig. 5b,f). The vessel densities in the wounds were assessed morphometrically after immunohistochemical staining for murine and human CD31. In both control and pre-vascularized substitutes, mouse-derived CD31^+^ vessels were localized at the border of substitutes at 7 days after grafting and distributed across the wounds at 14 days after grafting, indicating angiogenesis into the 3D skin substitutes. In contrast, in the pre-vascularized substitutes, engineered human-derived CD31^+^ vessels were consistently observed overall the substitutes during experiments (Fig. 6a). The vessel density was significantly higher in the pre-vascularized substitute-treated wounds than in the control-treated wounds (*p* < 0.01, Fig. 6b). Furthermore, immunofluorescence staining with human- and mouse-specific CD31 antibodies indicated the vessels formed by both engineered human- (green) and host-derived (red) endothelium at 14 days after grafting in the pre-vascularized substitutes (Fig. 6c).

**Figure 7:**
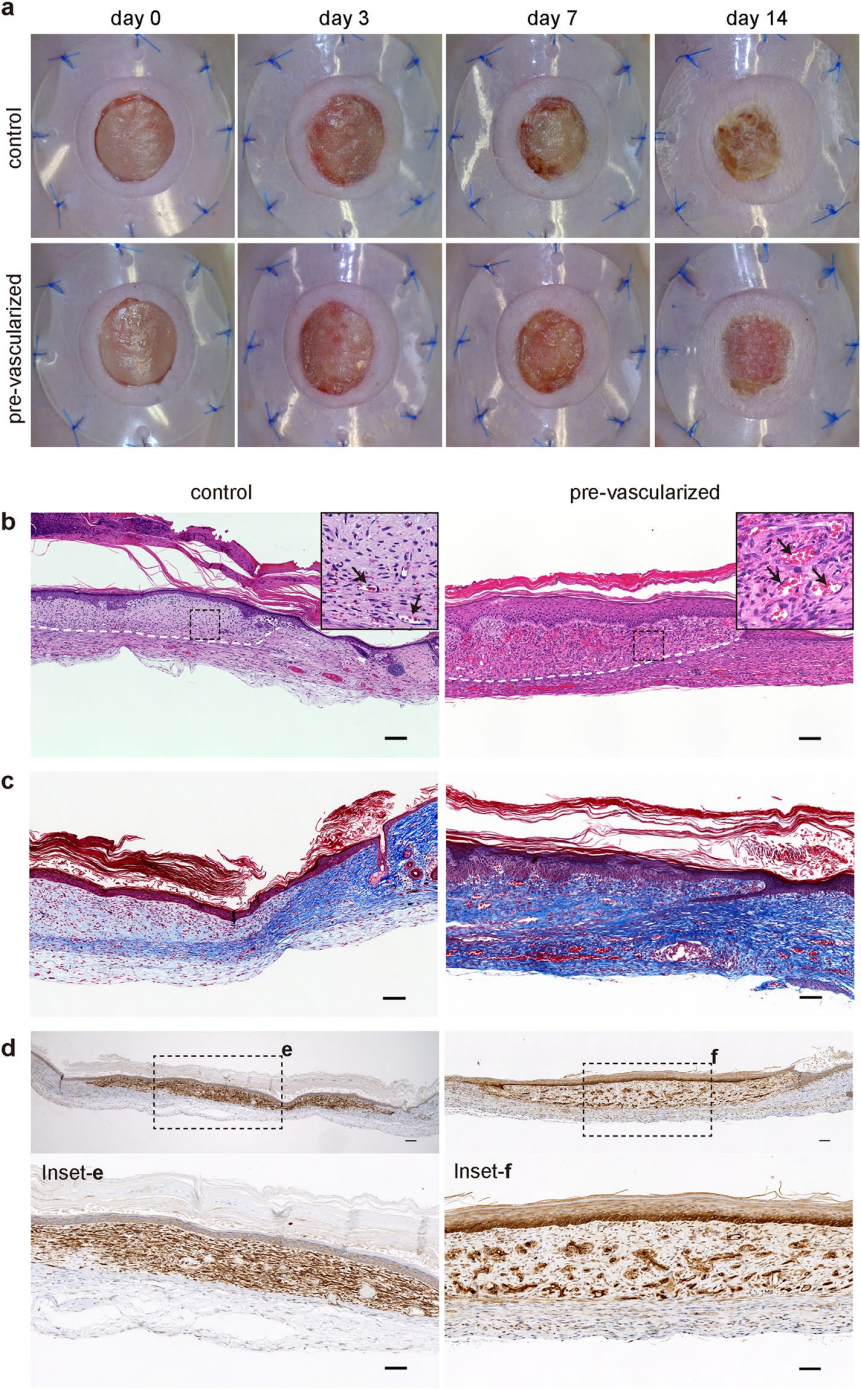
Pre-vascularization promoted wound healing and remodeling. (**a**) Macroscopic observation of transplanted control and pre-vascularized substitutes on the backs of immunodeficient mice up to 14 days post-transplantation. (**b**–**f**) Histological and immunohistochemical staining with hematoxylin and eosin (**b**), Masson’s trichrome (**c**), and HLA-ABC (**d**–**f**) of the wounds from the control and pre-vascularized substitutes at 14 days post-transplant. White dashed lines indicate the skin substitute–host interface. Black arrows indicate vessels perfused by mouse blood. Scale bars: 100 μm.

Visual observation in our *in vivo* grafting model showed that the control substitute-grafted sites exhibited exsiccation and detached from the wound site by day 14. Although 11 of 15 mice treated with non-vascularized control substitutes exhibited epidermolysis by day 7 after grafting (Fig. 5b), all control-treated wounds were completely re-epithelialized in mice at day 14 (Fig. 7b). In contrast, the pre-vascularized substitutes adhered to the wound tissue, which led to good healing with minimal wound contraction (Fig. 7a,b). Masson’s trichrome staining revealed mature collagen fiber (bright blue and densely packed) were observed in pre-vascularized substitutes, and the collagen level was similar to the host tissues. In contrast, the wounds of control-substitutes showed immature collagen (light blue and loosely packed) surrounded the fibroblasts (Fig. 7c). Moreover, we confirmed that the wound engraftment in mice was derived from the engineered human 3D skin substitutes by staining with human histocompatibility complex HLA-ABC. The engineered human vessels and Malpighian layer of pre-vascularized treated wound expressed uniformly HLA-ABC, indicating that the vasculature and epidermis are of human origin. In contrast, HLA-ABC positive cells in the control-treated wound were distributed on the dermis (Fig. 7d–f). Most importantly, the dermal and epidermal components derived from the grafted pre-vascularized substitutes survived 14 days post-transplantation, although the majority of control grafts were sloughed and immature.

## Discussion

In this study, we demonstrated that scaffold-free 3D skin substitutes with blood vessel networks were functional and reliable upon transplantation onto mouse full-thickness wounds. We successfully developed the novel skin substitutes using an LbL cell coating technique, which formed thin FN-G films on the cell surface. Our bioengineering approach made it possible to generate the pre-vascularized LbL 3D skin substitutes via approximately 2-week organotypic cultivation. This study is the first to demonstrate the potential application of scaffold-free pre-vascularized 3D skin substitutes for full-thickness skin defects.

Early excision of the wound eschar and subsequent coverage with skin substitutes are widely accepted as the primary goals in extensive burn treatment^19^. Bioengineered skin substitutes act first as protective dressings to limit infection and fluid loss, and further function as skin equivalents to provide temporary wound coverage or permanent skin replacement. Recently, various types of skin substitutes and techniques have become available to aid in wound coverage and healing^20^. However, even with increased research, the clinical application of tissue-engineered skin remains rare. One of the main reasons is the difficulty in vascularizing the tissue-engineered skin. Fibroblast-derived ECM components are essential for vascular formation and cell migration, adhesion, differentiation, and proliferation^21^. Recently, vascularization of skin constructs has been studied by seeding and cultivating vessel-forming cells on scaffolds^6,9,22,23^. FN-G coating of the cell surface by LbL assembly, which promotes cell-to-cell interaction and homogeneous stacking, is critical to the fabrication of the scaffold-free 3D-tissues^12^. In addition, coculturing the endothelial cells with FN-G-coated fibroblasts may enhance the formation of vasculature within a specific 3D tissue. In our study, vascularization was achieved in a short-term coculture system comprising endothelial cells and supportive FN-G-coated fibroblasts in the absence of exogenous materials such as scaffolds or growth factors. We feel that our unique approach has advantages over previous strategies for the generation of a human vessel network within a dermal layer.

In general, cultured human keratinocytes appear to promote optimal wound re-epithelialization and stimulate the regeneration of a compact neodermal component^24^. As an alternative to autologous split-thickness skin grafting, grafting of tissue-engineered skin substitutes composed of dermis and epidermis has been developed; the tissue-engineered skin substitutes act as a barrier, dermal matrix, and well-vascularized wound bed, and promote healing^25^. However, a lack of initial vascularization after grafting diminishes successful engraftment. Indeed, we clearly observed blood vessels only at the graft periphery, with limited numbers in the grafts, of non-vascularized 3D skin substitutes 7 days after grafting. In contrast, most of the pre-vascularized constructs were fully perfused 7 days after grafting. In addition, human vessel networks connect to host vasculature via the ingrowth of host vessels into the 3D skin substitutes at 14 days after grafting. The rapid perfusion of the grafted constructs by host blood in such a short time will likely alleviate tissue hypoxia and promote cell survival. As expected, we detected HLA-ABC^+^ cells in the grafted dermal and epidermal layers of the pre-vascularized substitutes, which indicated that the pre-vascularized 3D skin substitutes had a high graft survival rate. Shrinkage in *in vitro* human skin models is a critical drawback because it induces tissue necrosis, restricted graft survival, and faster degradation^26^. Indeed, a well-vascularized wound bed is essential for the survival of the skin graft and graft contraction. In this study, rapid perfusion also induced increases in collagen deposition and dermal thickness; we hardly observed graft contraction, which indicated that our pre-vascularized 3D skin substitutes are promising tools to promote the healing of full-thickness skin defects.

Although engrafted non-vascularized control substitutes have abundant fibroblasts, which secrete paracrine factors^27,28^, they undergo little neovascularization, resulting in a poor wound bed. In addition, epidermolysis occurred in the majority of non-vascularized substitutes at 7 days post-transplant, although they promoted complete re-epithelialization by 14 days. By contrast, we did not observe epidermal sloughing of the pre-vascularized 3D skin substitutes. Moreover, the wounds of control-substitutes showed that poor collagen deposition due to immature or inactive fibroblasts highly expressing HLA-ABC antigens, which may have been caused by an insufficient blood supply. The significantly higher vessel density in pre-vascularized substitutes at the early phase was associated with an enhanced maintenance, viability, and functional engraftment of 3D skin substitutes. Thus, our findings suggested that a preformed vasculature is a prerequisite for successful grafting.

Integration of the blood vasculature into tissue-engineered constructs can be crucial for the long-term viability and survival of the constructs *in vivo*^29^. In our strategy, the vascular network within the dermal layer was formed with a high proportion to HUVEC in the coculture system. However, endothelial cells were seeded at the lower density for the fabrication of the transplantable 3D skin because the grafts with a high number of HUVEC developed large subcutaneous and dermal hematomas. Hematomas can be a source of infection, so skin substitutes must be designed to prevent wound hematomas. Although the rapidity of graft vascularization is important for protection against infection to facilitate effective skin graft integration, future studies must address other factors that affect the potential of our approach for enhancing tissue survival and resistance against wound infection.

In summary, we demonstrated the potential of scaffold-free pre-vascularized LbL 3D skin as an implantable tissue substitute. The tissue constructs comprising endothelial cells and a high density of FN-G-coated fibroblasts promoted the development of vessel networks *in vitro*, accelerated the rate of anastomosis with host vessels, and aided healing *in vivo*. Our strategy to transplant a dermo-epidermal skin substitute containing blood vessels may facilitate the efficient reconstruction of full-thickness skin defects.

## Methods

### Cell culture

Neonatal normal human dermal fibroblasts (NHDF, CC-2509; Lonza, Basel, Switzerland) were cultured in Dulbecco’s modified Eagle’s medium (DMEM, Nacalai Tesque, Kyoto, Japan) supplemented with 10% fetal bovine serum (FBS), 100 U/mL penicillin, 100 µg/mL streptomycin, and 0.25 µg/mL amphotericin B at 37 °C in 5% CO_2_. Human umbilical vein endothelial cells (HUVEC; Lonza) and GFP-HUVEC (Angio-Proteomie, Boston, MA, USA) were cultured in endothelial cell medium (EGM-2MV, Lonza). After reaching 80–90% confluency, the NHDF and HUVEC were subcultured using 0.25% trypsin/EDTA solution (Lonza) and used at passage 5 to construct the 3D skin models. Neonatal human epidermal keratinocytes (HEKn, Gibco, Carlsbad, CA, USA) were cultured in EpiLife® medium with 60 μM calcium (Invitrogen, Carlsbad, CA, USA) supplemented with HuMedia-KG growth factor (KURABO, Osaka, Japan). Media were changed every other day.

### Preparation of vascularized 3D skin substitutes

The 3D skin containing the vascular network was constructed via an LbL technique that enabled the coating of individual cells with FN-G ECM films on NHDF surfaces without damage. Briefly, NHDF were alternately incubated for 1 min with 0.04 mg/mL fibronectin (FN) or gelatin (G) solutions in phosphate-buffered saline (PBS) and rinsed with PBS between each coating step. The coating procedures were performed a total of 9 times (FN: 5 times; G: 4 times). Then, to construct the dermal layer, FN-G-coated NHDF (viability >97%, 1 × 10^7^ cells/insert) and HUVEC (0.1 × 10^5^, 0.2 × 10^5^, or 1 × 10^5^ cells/insert) or FN-G-coated NHDF alone were seeded onto 12-well culture inserts (1.12-cm^2^ membrane growth area, 0.4-μm pores; Corning Life Science, Tewksbury, MA, USA) pre-coated with FN; the inserts were cultured in DMEM containing 5% FBS for 24 hours. In other experiments, GFP-HUVEC were seeded to facilitate the monitoring of vessel formation during the cultures. After 24 hours coculture of FN-G-coated NHDF and HUVEC, type IV collagen solution (from human placenta, 0.2 mg/mL in PBS; Sigma-Aldrich, St. Louis, MO, USA) was dropped onto the upper surface of the dermis, and the constructs were incubated for 30 min. Subsequently, HEKn (passage 4, 1 × 10^6^ cells/insert) were seeded onto the collagen type IV-coated dermal layer, then cultured for 24 hours in growth medium (5% FBS/DMEM and EpiLife^®^ at a ratio of 1:1), and air–liquid interface-cultured for 7 days to drive epidermal differentiation, stratification, and cornification in growth media supplemented with 25 μg/mL ascorbic acid. The medium in the lower chamber was replaced every day; flooding of the top chamber was avoided. For *in vivo* experiments, we seeded 0.2 × 10^5^ HUVEC per construct.

### Cell viability assay in 3D skin substitutes

We determined cell viability in the 3D skin substitutes using the LIVE/DEAD Viability/Cytotoxicity kit (L-3224; Molecular Probes, Eugene, OR, USA) according to the manufacturer’s protocol. Briefly, at 2, 4, and 7 days after organotypic coculture, we rinsed the 3D skin substitutes (n = 3 for each time point) 3 times with PBS, then stained with 2 μM calcein AM (which stained live cells green) and 0.5 μM ethidium homodimer (which stained dead cells red) in PBS for 30 min at room temperature. After incubation, the samples were washed twice with PBS and imaged immediately using a Biozero BZ-X700 fluorescence microscope (Keyence, Osaka, Japan).

We acquired 3 images of different portions of each triplicate sample for each time point. Still images of the middle section of the 3D skin substitute (~100 to 120 µm from the culture insert membrane) were captured and serial micrographs were later combined for z-stacked compilation images. The experiment was performed twice.

### Transplantation of tissue-engineered 3D skin substitutes

All surgical procedures were conducted according to protocols approved by the National Defense Medical College Animal Care and Use Committee (Permit number: 17012). Pre-vascularized 3D skin substitutes were transplanted to excisional wounds in C.B-17 SCID mice. Eight-week-old male mice (body weight, 24.2 ± 3.7 g; Charles River Laboratories, Kanagawa, Japan) were randomly divided into 3 groups (n = 15 animals/group). The excisional wound splinting model was employed as previously described^30^. In brief, mice were anesthetized with an intraperitoneal injection of ketamine (100 mg/kg) and xylazine (10 mg/kg), then their dorsal surfaces were shaved with electric clippers and treated with a depilatory agent to remove remaining hair. Under sterile conditions, a circular full-thickness excisional wound (8-mm diameter) was created on the middle dorsum of each mouse using a biopsy punch. A donut-shaped silicone splint (0.5-mm thick, 12-mm inner diameter, 20-mm outer diameter) was placed around the perimeter of the wound and fixed to the skin with cyanoacrylate glue (Aron Alpha, Toagosei, Tokyo, Japan) and interrupted 6-0 nylon sutures (Fig. 1). The splint was used to minimize wound contraction. The skin substitutes were cut according to the dimensions of the full-thickness wound, and each wound was treated with a different substitute: pre-vascularized 3D skin, non-vascularized 3D skin (negative control), or acellular synthetic bilayer skin, which is a collagen-glycosaminoglycan sponge with a temporary silicone rubber epidermal barrier. The grafts were covered with a non-adherent silicone gel dressing (SI-AID, ALCARE, Tokyo, Japan) and wrapped with a semipermeable adhesive film (OPSITE, Smith & Nephew, Largo, FL, USA) to protect the wound site and prevent desiccation. The animals were housed individually. After 7 (n = 7/group) or 14 (n = 8/group) days, the mice were euthanized and tissue biopsies of the grafted areas were collected.

### Transillumination stereomicroscopy

To visualize the vascular network by transillumination, the implants and surrounding skin were excised at day 7 after grafting; specimens were then quickly placed on a Petri dish and observed under an inverted microscope.

### Histology and immunostaining

To observe the vessel network formed by HUVEC *in vitro* after 7 days of culture, we stained whole-mount 3D skin substitutes with a fluorescently labeled anti-CD31 antibody. The constructs (n = 3) were fixed in 4% paraformaldehyde for 15 min, then permeabilized in PBS with 0.2% Triton™ X-100 for 15 min. The tissues were blocked with 1% bovine serum albumin for 1 h, then incubated with a mouse anti-human CD31 antibody (1:100; M0823, clone JC70A; Dako, Carpinteria, CA, USA). The tissues were incubated with Alexa Fluor® 546-conjugated goat anti-mouse IgG (1:500; Invitrogen) for 1 h. Immunofluorescence images of the stained vessels were taken with a BZ-X700 microscope. Vascular profiles were characterized by positive staining for human CD31 in structures with an identifiable vascular lumen (>5 μm). The vascular area density and number of branching points were analyzed with AngioTool software (National Cancer Institute, Rockville, MD, USA). All analyses were carried out manually.

The grafted sites were fixed in 10% neutral buffered formalin overnight, then dehydrated and embedded in paraffin. Hematoxylin and eosin, trichrome, and immunohistochemical/immunofluorescent staining were performed on 4-μm-thick tissue sections. Trichrome staining was carried out using the Modified Gomori’s One-Step Trichrome Staining Kit (Biocare Medical, Concord, CA, USA) and immunohistochemical staining was performed with the Vector ImmPRESS™ peroxidase polymer Anti-mouse or Anti-rabbit IgG reagent kits (Vector Labs, Burlingame, CA, USA) according to the manufacturers’ instructions. For immunofluorescent staining, we incubated with Alexa Fluor® 488-conjugated (Abcam, Cambridge, MA, USA) and Alexa Fluor® 594-conjugated (Molecular Probes, Eugene OR, USA) secondary antibodies, then performed nuclear staining with 4′,6-diamidino-2-phenylindole (Sigma-Aldrich, St. Louis, MO, USA). The following primary antibodies were used for immunostaining of: a human–specific CD31 (1:200, NBP2-15202, clone C31.3; Novus Biologicals, Centennial, CO, USA), a mouse–specific CD31 (1:100, 14-0311-82, clone 390; Invitrogen), HLA-Class I ABC (1:2,500, ab70328, clone: EMR8-5; Abcam), and laminin 5 (1:200; ab14509, Abcam). The MOM kit (Vector Labs) was used for mouse derived monoclonal antibodies.

### Statistical analysis

The data are expressed as the mean ± standard deviation (SD). Statistical analyses were performed by unpaired Student’s *t*-test or one-way analysis of variance (ANOVA) with Tukey’s multiple comparison post hoc test using GraphPad Prism 7.0 software (La Jolla, CA, USA). P-values < 0.05 were considered significant.
